# An extended catalog of integrated prophages in the infant and adult fecal microbiome shows high prevalence of lysogeny

**DOI:** 10.3389/fmicb.2023.1254535

**Published:** 2023-09-05

**Authors:** Evgenia Dikareva, Dollwin Matharu, Emilia Lahtinen, Kaija-Leena Kolho, Willem M. De Vos, Anne Salonen, Alise J. Ponsero

**Affiliations:** ^1^Human Microbiome Research Program, Faculty of Medicine, University of Helsinki, Helsinki, Finland; ^2^Children's Hospital, Paediatric Research Centre, University of Helsinki and HUS, Helsinki, Finland; ^3^Faculty of Medicine and Health Technology, Tampere University, Tampere, Finland; ^4^Laboratory of Microbiology, Wageningen University and Research, Wageningen, Netherlands

**Keywords:** infant gut microbiota, bacteriophages, prophage, lysogeny, metagenome assembled genomes (MAGs), auxilliary metabolic genes

## Abstract

**Background and aims:**

The acquisition and gradual maturation of gut microbial communities during early childhood is central to an individual’s healthy development. Bacteriophages have the potential to shape the gut bacterial communities. However, the complex ecological interactions between phages and their bacterial host are still poorly characterized. In this study, we investigated the abundance and diversity of integrated prophages in infant and adult gut bacteria by detecting integrated prophages in metagenome assembled genomes (MAGs) of commensal bacteria.

**Methods:**

Our study included 88 infants sampled at 3 weeks, 3 months, 6 months, and 12 months (*n* = 323 total samples), and their parents around delivery time (*n* = 138 total samples). Fecal DNA was extracted and characterized by using shotgun metagenomic sequencing, and a collection of prokaryotic MAGs was generated. The MAG collection was screened for the presence of integrated bacteriophage sequences, allowing their taxonomic and functional characterization.

**Results:**

A large collection of 6,186 MAGs from infant and adult gut microbiota was obtained and screened for integrated prophages, allowing the identification of 7,165 prophage sequences longer than 10 kb. Strikingly, more than 70% of the near-complete MAGs were identified as lysogens. The prevalence of prophages in MAGs varied across bacterial families, with a lower prevalence observed among *Coriobacteriaceae, Eggerthellaceae, Veillonellaceae and Burkholderiaceae*, while a very high prevalence of lysogen MAGs were observed in *Oscillospiraceae, Enterococcaceae, and Enterobacteriaceae*. Interestingly for several bacterial families such as *Bifidobacteriaceae* and *Bacteroidaceae*, the prevalence of prophages in MAGs was higher in early infant time point (3 weeks and 3 months) than in later sampling points (6 and 12 months) and in adults. The prophage sequences were clustered into 5,616 species-like vOTUs, 77% of which were novel. Finally, we explored the functional repertoire of the potential auxiliary metabolic genes carried by these prophages, encoding functions involved in carbohydrate metabolism and degradation, amino acid metabolism and carbon metabolism.

**Conclusion:**

Our study provides an enhanced understanding of the diversity and prevalence of lysogens in infant and adult gut microbiota and suggests a complex interplay between prophages and their bacterial hosts.

## 1. Introduction

During and after birth, the newborn gut is rapidly colonized by commensal microbes, and the gut microbiota develops in infancy into a complex population of prokaryotes, micro-eukaryotes, and viruses. The virome is composed of both viruses infecting bacteria (bacteriophages) and eukaryotic viruses, with the phages constituting most of the diversity and abundance of the gut virome (Shamash and Maurice, 2022). The acquisition of the gut microbiome is critical for the infant immune and physiological development and disruptions in the gut microbiota during this early developmental window can have long-lasting health consequences (Gensollen et al., 2016; Stiemsma and Michels, 2018). Phages have been shown to be major drivers shaping bacterial communities in many ecosystems, both in a lytic and/or temperate manner (Fuhrman, 1999; Suttle, 2007; Trubl et al., 2018; Tran and Anantharaman, 2021). While the dynamics of the bacterial colonization and maturation have been extensively studied from infancy to adulthood, the role and importance of phages in shaping early life gut microbiota development is relatively understudied (Mirzaei and Maurice, 2017; Dahlman et al., 2021).

It is estimated that in the healthy adult gut there is a phage-to-bacteria ratio of approximately 1:1 or as low as 0.1:1 (Kim et al., 2011; Hoyles et al., 2014). This number is much lower than the 10:1 ratio estimated in marine ecosystems (Wigington et al., 2016), where a ‘kill-the-winner’ dynamic is observed, in which lytic phages play a central role in shaping and controlling bacterial populations. On the other hand, in the gut microbiome, the relatively high abundance of lysogenic phages and the low phage-to-bacteria ratio suggest a very different dynamic and a prevalence of temperate life cycle (Reyes et al., 2012; Silveira and Rohwer, 2016), in which the phages integrate their genome into host chromosomes as prophages. Strikingly, classic virome approaches tend to neglect integrated prophages, as they typically enrich viral-like particles by filtering out cells and free cellular DNA from the samples, therefore missing prophages integrated in their bacterial host. However, recent studies and computational tools have enabled the mining of bulk metagenomes for novel phage sequences and provide exciting new avenues to explore phage communities directly in their environment (Hurwitz et al., 2018). Indeed, genome-resolved metagenomics approaches allow for the reconstruction of metagenome assembled genomes (MAGs), enabling the characterization of functional potential and genome comparison at a finer scale for individual bacterial taxa. Importantly, MAGs enable also the characterization of prophage – bacterial host relationships (Nayfach et al., 2021c; Johansen et al., 2022; Tomofuji et al., 2022).

Between 30 and 75% of all complete sequenced bacterial genomes independent of the ecosystem contain one or more prophage sequence (Casjens, 2003; Roux et al., 2015; López-Leal et al., 2022), and they have been shown to modulate their host fitness by several mechanisms (Howard-Varona et al., 2017). In particular, prophages can affect the host cell’s physiology by introducing novel functions or modulating pre-existing ones, such as virulence factors, metabolism genes and immunity to phages (Hargreaves et al., 2014). Prophages can therefore encode additional metabolic genes that are not required for the phage life cycle but rather augmenting the hosts’ metabolism, providing a benefit for phage-infected versus non-infected bacteria within a given ecosystem (Brown et al., 2022). These auxiliary metabolic genes (AMGs) include genes involved in cell survival and growth, nutrient uptake systems but also defensive and offensive factors (e.g., toxins). While the diversity and potential impact of AMGs have been explored in marine and soil viral communities, their impact on human microbiota is largely unexplored. Prior efforts in adult fecal phageome suggested the presence of potential AMGs involved in the anaerobic synthesis of nucleotides and proteins involved in oxidative stress response (Reyes et al., 2010), as well as carbohydrate-active enzymes, peptidases, carbon-and nitrogen-metabolisms (Shaffer et al., 2020).

Previous studies have explored the dynamics and diversity of the infant gut virome and suggested the importance of temperate phage lifestyle in infant gut microbiome (Lim et al., 2015; Bushman and Liang, 2021; Shah et al., 2023; Walters et al., 2023). However, the specific prevalence and diversity of integrated prophages in infant gut remains largely unexplored, and to our knowledge, the diversity and potential role of viral AMGs in the infant gut is still uncharacterized. To address this gap, we generated a collection of 6,186 MAGs assembled from 461 infant and adult fecal metagenomic samples from the Health and Early Life Microbiota (HELMi) birth cohort (Korpela et al., 2019). We mined these medium and high-quality MAGs for potential integrated prophage sequences, in order to assess the prevalence, diversity and novelty of integrated prophages detected in these MAGs. Finally, we explored the functional potential of the putative AMGs carried by these prophages.

## 2. Materials and methods

### 2.1. Sample collection and sequencing

The HELMi birth cohort study (*N* = 1,055) is a prospective follow-up study on early life microbiota and health (Korpela et al., 2019) NCT03996304. For this study, 88 infants were included. Fecal samples collected at age of 3 weeks, 3, 6 and 12 months, and the maternal and paternal samples collected around delivery time: from 3 weeks before delivery up to 3 months after delivery for maternal samples (median = 8 days before delivery, IQR (interquartile range) = 8) and from 3 weeks before delivery to 15 months after delivery for paternal samples (median = 5 days before delivery, IQR = 7,75). The samples were collected at home and stored immediately at −20°C until being transported frozen to the lab, for long term storage at −80°C. The study was approved by the ethical committee of The Hospital District of Helsinki and Uusimaa and performed in accordance with the principles of the Helsinki Declaration. Parents signed an informed consent at enrolment.

DNA was extracted from the stool samples using a bead-beating method. In short, approximately 250 or 340 mg of faecal material was suspended in 0.5 or 1 mL of sterile ice-cold PBS, and 250 μL of the faecal suspension was combined with 340 μL of RBB lysis buffer (500 mM NaCl, 50 mM Tris–HCl (pH 8.0), 50 mM EDTA, 4% SDS) in a bead-beating tube from the Ambion MagMAX™ Total Nucleic Acid Isolation Kit (Life Technologies). After repeated bead-beating, 200 μL of the supernatant was used for DNA extraction with a KingFisherTM Flex automated purification system (ThermoFisher Scientific) using a MagMAXTM Pathogen High Vol. DNA was quantified using Quanti-iT™ Pico Green dsDNA Assay (Invitrogen).

Sequencing libraries were prepared according to the Nextera DNA Flex Library Prep Reference Guide (v07) (Illumina, San Diego, CA, USA), but the reaction volumes were scaled down to ¼ of the protocol volumes. Sequencing was performed with the Illumina NovaSeq system using S4 flow cells with lane divider (Illumina, San Diego, CA, USA) at the sequencing laboratory of the Institute for Molecular Medicine Finland FIMM Technology Centre, University of Helsinki. Each pool was sequenced in a single lane, using a read length for the paired-end run was 2 × 151 bp.

### 2.2. Quality control, human read filtering and read annotation

Quality control (QC) and removal of human sequences were performed using fastqc v0.11.9 and trimGalore v0.6.6 with default parameters (Krueger, 2015). Quality-filtered sequences were screened to remove human sequences using bowtie2 v2.4.2 (Langmead and Salzberg, 2012) against a non-redundant version of the Genome Reference Consortium Human Build 38, patch release 14.^1^

All samples had a minimum of 10 million paired reads after QC and human filtering. Taxonomic profiling at the read level was performed using Kraken2 (Wood and Salzberg, 2014) and Braken (Lu et al., 2017). Kraken2 v2.1.1 was run on the paired read against the HumGut database (Hiseni et al., 2021), and Bracken v2.6.1 was run on the Kraken2 outputs.

Human sequence-filtered raw reads are accessible at ENA (Study ID: PRJEB52774). The ENA ID of each run used in this project is listed in Supplementary File 1.

### 2.3. Metagenome assembled genomes (MAGs)

After QC and removal of human sequences, the reads were assembled using Megahit v1.2.9 (Li et al., 2015). Metagenomes generated from the same infant were co-assembled, while parental samples were assembled independently. The assembled contigs were then used to obtain MAGs using the MetaWRAP pipeline v1.3 (Uritskiy et al., 2018). Briefly, this pipeline leverages MaxBin v2.2.7 (Wu et al., 2016), MetaBAT2 v2.15 (Kang et al., 2019) and CONCOCT v1.1.0 (Alneberg et al., 2014). After a bin refinement step (comparison of the bins obtained by the different binning tools and selection of the bins with higher completion and lower contamination), the bin quality was assessed using CheckM v1.12 (Parks et al., 2015), and bins with a minimum of 70% completion and a maximum of 5% contamination were selected. The selected bins were reassembled, and their quality assessed with CheckM. The aim of this reassembly step is to improve the original set of bins, by mapping the samples reads to the bin, reassembling the contigs and evaluating the reassembled bin quality using CheckM. The best bin obtained (original or reassembled) was kept for the rest of the analysis. The obtained MAGs were clustered using dRep v3.4.2 (Olm et al., 2017) using an ANI threshold of 95% and a coverage threshold of 50%. Finally, the MAGs taxonomic classification was obtained using GTDB-tk v2.3.0 (Chaumeil et al., 2022).

The abundances of each MAG present in each sample were calculated using the Quant_bin module from MetaWRAP, which leverages Salmon (Patro et al., 2017) to estimate the abundance of each scaffold in each sample, and then computes the average MAG abundances, expressed as genome copies per million reads. The infant MAGs were categorized into “Early” and “Late” categories according to their difference in abundance at the early sampling points (3 weeks and 3 months) and later infant sampling point (6 and 12 months).

The fasta sequence of the assembled MAGs from this study are available in the Zenodo repository 10.5281/zenodo.8063476.

### 2.4. Prophage detection and classification

Putative prophage sequences were identified on the HELMi MAGs using Virsorter2 v2.2.4 (Guo et al., 2021). We leveraged the prophage boundaries identified by VirSorter2 to remove the potential bacterial host sequences located up and downstream of the integrated prophage sequence. The predicted prophage sequences were further screened for false positives using CheckV v1.0.1 (Nayfach et al., 2021a). Putative prophage sequences longer than 10 kb, classified as prophages by VirSorter2 and/or CheckV, and with at least 1 phage gene hit as well as contigs with no cellular hits confirmed by CheckV were kept for further analysis. The fasta sequence of the prophages identified in this study are available in the Zenodo repository 10.5281/zenodo.8063476.

Prophage sequences were dereplicated using mmSeqs2 v14 (Steinegger and Söding, 2017) using a threshold of 99% ANI. Species-like vOTUs were obtained using mmSeqs2 using a threshold of 95% ANI over 75% of the shortest sequence, as previously described (Li et al., 2022), and genus-like vOTUs were predicted using vContact2 v0.11.3 (Bin Jang et al., 2019), as previously described (Li et al., 2022). The HELMi prophage sequences were compared to previously published phage sequences from 5 databases and catalogues focusing on phage sequences from human gut metagenomes:

- The Gut Virome database (GVD version 1; *n* = 33,242 reference sequences) (Gregory et al., 2020)
- The Metagenomic gut virus (MGV; *n* = 54,118 reference sequences) (Nayfach et al., 2021b)
- The Cenote human virome database (CHVD; *n* = 45,033 reference sequences) (Tisza and Buck, 2021)
- The Gut Phage database (GPD; *n* = 142,809 reference sequences) (Unterer et al., 2021)
- IMG/VR vOTUs from human gut ecosystems (*n* = 63,424 reference sequences) (Camargo et al., 2023a)
- COPSAC infant phages (*n* = 10,021 reference sequences) (Shah et al., 2023)
- Gut Phages from Benler et al. (*n* = 3,738 reference sequences) (Benler et al., 2021)
- Japanese 4D catalog (*n* = 1,347 reference sequences) (Nishijima et al., 2022)
- Danish Enteric Virome Catalog (DEVoC) (*n* = 12,986 reference sequences) (Van Espen et al., 2021)

The reference sequences from these catalogues and databases were dereplicated and clustered into species-like vOTUs as described above. The HELMi prophage sequences were compared to the catalog by clustering the sequences at 95% ANI over 75% of the shortest sequence.

Family-level phage taxonomy was predicted using PhaGCN (Shang et al., 2021) on the species-like vOTUs leveraging the updated ICTV taxonomic classification model and taxonomic names.^2^ Genomad (Camargo et al., 2023b) was used to obtain Class-level phage annotation of the vOTUs, using a database downloaded in May 2023.

### 2.5. Functional annotation of prophage sequences

Putative viral auxillary metabolic genes (pAMGs) were predicted on the prophage contigs longer than 10 kb using the DRAM-v module from DRAM v1.4.6 (Shaffer et al., 2020). The tool annotates each reading frame using several viral and metabolic reference databases including KEGG, PFAM and CAZY, and generates a list of potential AMGs. To avoid reporting potential false positives, pAMG were only considered for metabolic genes located on prophage contig with a “Possible non-viral contig score” defined by DRAM-v below 0.25. Additionally, we considered only metabolic genes situated between at least 2 phage genes (DRAM-v AMG score 1, 2, 3) and at more than 5 kb from the contig end without any transposon at proximity.

We further manually checked pAMGs with glycosyl hydrolases, glycosyl transferases and polysaccharide lyase annotations. We only considered pAMGs located in a prophage sequence qualified as “Complete” or “High-quality” by CheckV. The pAMG protein sequence was submitted to HHpred (Zimmermann et al., 2018) against the PDB_mmCIF70_17_Apr database, and pAMG without a significant and concordant hit (probability<90%) were excluded. The genetic context of the pAMG was also manually inspected to ensure the presence of reasonable phage hits up and downstream of the pAMG on the contig sequence.

## 3. Results

### 3.1. Recovering bacterial MAGs from infant and adult gut microbiota

**Figure 1:**
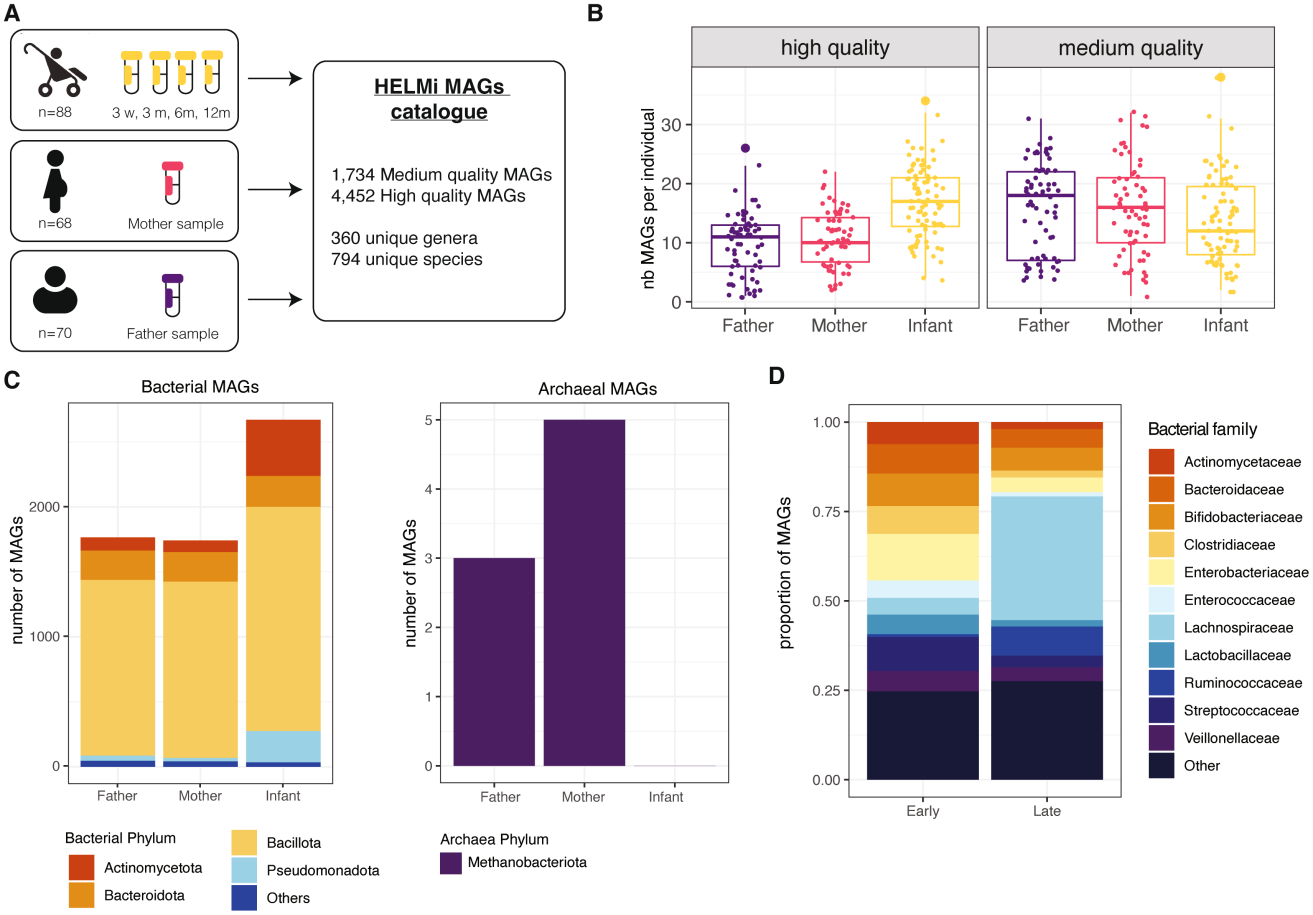
HELMi MAG collection overview. **(A)** HELMi metagenome-assembled genome (MAG) collection overview. The samples from infants were collected at 4 time points and co-assembled per infant before binning. Parental samples were collected around delivery time, and each sample was assembled individually and binned. Bins with a completeness above 70% and contamination below 5% were considered a MAG and taxonomically annotated. **(B)** Number of MAGs obtained per individual. MAGs were further classified into “High quality” (>90% completeness and < 5% contamination) and “Medium quality (>70% completeness and < 5% contamination) as assessed using CheckM. The number of each category of MAGs obtained per individual (infant, mother or father) was computed. **(C)** Taxonomic classification of the MAGs. Taxonomic classification of the high quality and medium quality MAGs was obtained using GTDB-tk. Counts are aggregated at the phylum level and archaeal and bacterial MAGs are represented separately. **(D)** Taxonomic classification of infant MAGs. HELMi MAGs assembled from infants’ samples were classified as “Early” and “Late” according to their relative abundances in “early” (3 weeks and 3 months) and “late” (6 and 12 months) sampling time points. Taxonomic classification of the high quality and medium quality MAGs was obtained using GTDB-tk. Counts are aggregated at the family level.

The present study includes a subset of 90 families from the broader HELMi cohort (NCT03996304) (Korpela et al., 2019). All infants were born in Finland in hospital at term and followed up for one year. Infant stool samples were collected at 3 weeks (*n* = 86), 3 (*n* = 86), 6 (*n* = 87) and 12 months (*n* = 64) of age and parental samples (*n* = 68 maternal and *n* = 70 paternal samples) were collected from 3 weeks before the delivery up to 15 months after delivery (mothers’ samples: median = −8 days, IQR = 8; fathers’ samples: median = −5 days, IQR = 7.75). The cohort’s general characteristics are summarized in Supplementary Table 1. In total, 461 samples were sequenced using shotgun metagenomic sequencing and assembled to obtain Metagenome Assembled Genomes (MAGs) from the prokaryotic fraction of the microbiota (Figure 1A). For this study, we considered a bin to qualify as a MAG if its completeness was above 70% and its contamination below 5%. This strategy produced 1,734 medium quality MAGs defined here by a completeness ranging from 70 to 90% with contamination below 5% and 4,452 high-quality MAGs defined by a completeness above 90% with an estimated contamination below 5% (Bowers et al., 2017). The number of high-quality MAGs retrieved per sample was higher for metagenomes from infants than adults, as the samples collected from the same infants were co-assembled (Figure 1B).

Using pair-wise average nucleotide identities (ANI) comparisons, we assessed the redundancy of the HELMi MAGs. With a cut-off of 95% ANI and a coverage above 50%, the MAGs could be clustered into 1,172 unique clusters, suggesting a relatively high redundancy of the catalog. The HELMi MAGs were taxonomically annotated using GTDB-tk (Chaumeil et al., 2022). All the 6,681 MAGs were classified to the family level, 6,183 (99.9%) to the genus level and 6,089 (98.4%) to the species level. As expected from stool samples, a high proportion of MAGs from the bacterial phyla Bacillota (previously Firmicutes *n* = 4,436, 71.7%), Bacteroidota (previously Bacteroidetes, *n* = 689, 11.1%) and Actinomycetota (previously Actinobacteria, *n* = 626, 10.1%) were retrieved in both parents and infants (Figure 1C). Only 8 MAGs from the archaea *Methanobrevibacter smithii* were retrieved from parental samples (Figure 1C). The composition of the HELMi MAG catalog corresponds to the global average composition of the samples observed at the read level (Supplementary Figure 1A). In total, the HELMi MAG catalog contains MAGs from 794 distinct species and 360 distinct genera, and 197 unique species (55%) were found in both parental and infant derived MAGs.

The sequencing reads were mapped to the MAGs assembled from the sample to calculate the relative abundance of each MAG in their respective samples. As expected, the MAGs retrieved accounted for taxa found in abundance in the samples, suggesting that only the main bacterial taxa of the communities are represented in this catalog (Supplementary Figure 1B). The MAGs generated from infant samples were classified into “Early” and “Late” categories according to their difference in relative abundance in early (3 weeks and 3 months) and later infant sampling point (6 and 12 months). The “Early” MAGs category included mostly MAGs from *Enterobacteriaceae, Streptococcaceae, Bifidobacteriaceae* and *Bacteroidaceae* bacterial families, while the “Late” MAG category included a larger number of MAGs from *Lachnospiraceae* and *Ruminococcaceae* bacterial families (Figure 1D). The total list of MAGs generated along with their quality and taxonomic annotations are available in Supplementary File 2.

### 3.2. Most bacterial MAGs assembled from infant and adult gut contain prophage sequences

We screened the HELMi MAGs for potential prophage sequences using VirSorter2 (Guo et al., 2021), and the putative prophage sequences were confirmed using CheckV (Nayfach et al., 2021a). In order to exclude highly degraded phages, we only considered sequences longer than 10 kb with detected bacterial host flanking sequences. A total of 7,165 predicted prophages were retrieved by this approach, from which 787 were complete phage genomes, 755 high-quality sequences, and 1,872 medium quality sequences as assessed by CheckV. The prophage sequences and their characteristics are summarized in the Supplementary File 3.

**Figure 2:**
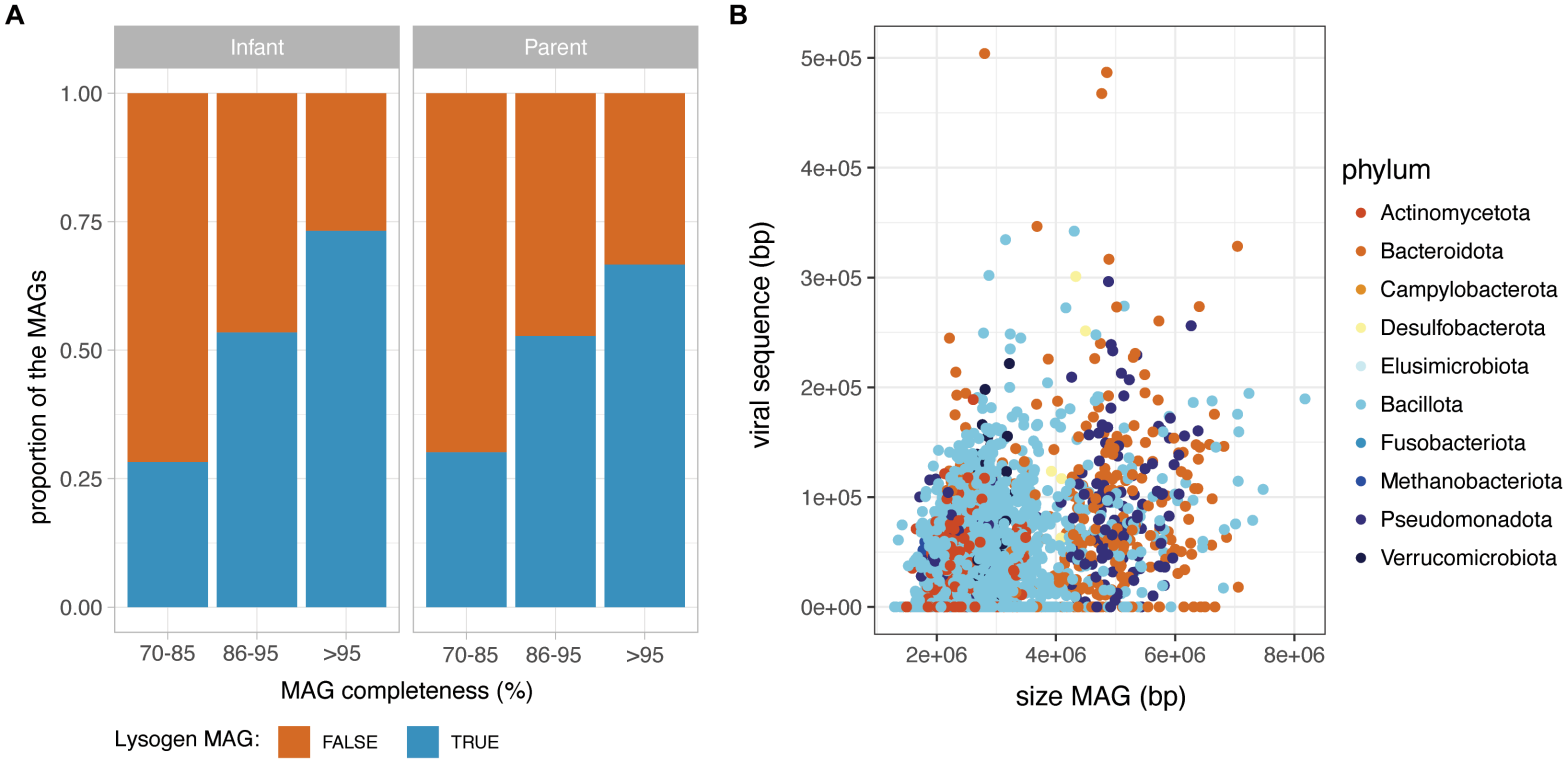
Prophage content in the HELMi MAG catalogue. **(A)** Prevalence of lysogen MAGs in infants and adult samples. MAGs were aggregated according to their MAG completeness as assessed by CheckM, and classified as lysogens or non-lysogens according to their content in prophages. **(B)** Relationship between MAG length and total prophage sequence length in infants and adult MAGs. MAGs were subsetted to include only high-quality MAGs (above 95% completeness).

Importantly, the MAGs without detectable phage sequence had a significantly lower completeness than MAGs with at least one prophage detected (Wilcoxon, *p* < 0.05), suggesting that the absence of detected prophages in incomplete MAGs is likely due to a technical limitation. Therefore, to investigate the prevalence of lysogen MAGs (defined as a MAG with a least one detectable prophage), they were considered according to their completeness range (70–85%; 86–95 and > 95% completeness). For both infant and adult MAGs, the proportion of lysogens increased sharply with MAG completeness, with a proportion of lysogens in near-complete MAGs (>95% completeness), reaching 74% in infants and 67% in parental MAGs (Figure 2A). On the subset of 2,985 near-complete MAGs (>95% completeness), the total length of prophage content per MAG was weakly correlated to the total size of the MAG (Spearman rank correlation rho = 0.30, value of *p*<2.2e-16) (Figure 2B), but not to the MAG completeness (Spearman rank correlation rho = 0.075, value of *p*<0.05). Prophage sequences accounted for 1.1% in median of the total MAG sequences (IQR = 2.1) and the proportion of prophage sequences in MAGs was globally consistent across bacterial families (Supplementary Figure 2).

**Figure 3:**
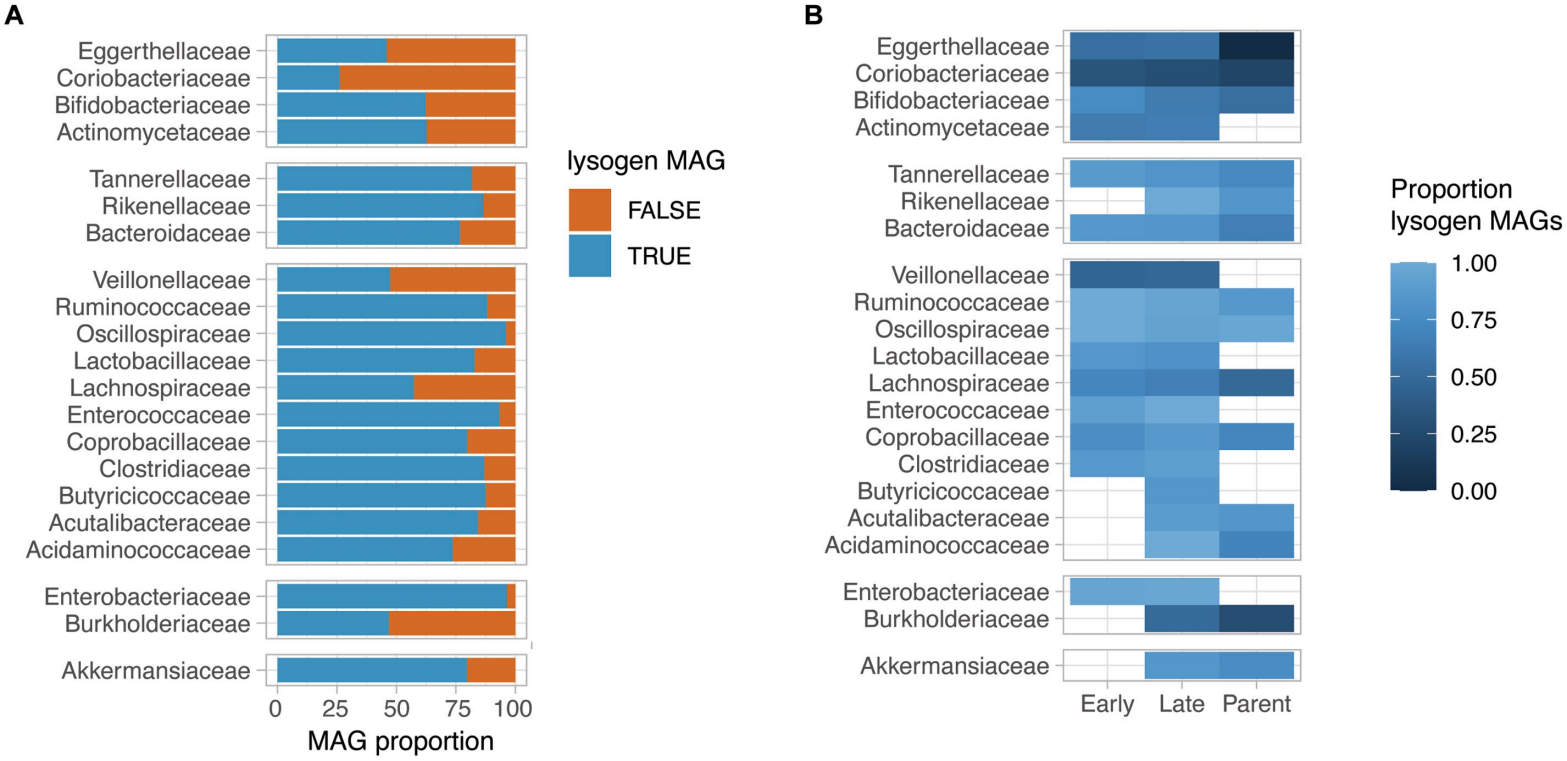
Lysogeny prevalence in HELMi MAGs. **(A)** Proportion of lysogen MAGs per bacterial family. We selected bacterial families for which more than 30 near-complete MAGs (>95% completeness) were available. A lysogen MAG was defined as a MAG for which at least one prophage sequence was detected. Bacterial families are grouped by phylum. **(B)** Proportion of lysogen MAGs per bacterial family and MAG groups. We selected bacterial families for which more than 30 near-complete MAGs (>95% completeness) were available. The MAGs assembled from infant samples were categorized into “Early” and “Late” categories according to their relative abundances at early (3 weeks and 3 months) and late (6 and 12 months) sampling time points. The proportion of lysogen MAGs (MAG with at least one detectable prophage sequence) was calculated independently per bacterial families and MAG categories for groups containing at least 5 MAGs. Bacterial families are grouped by phylum.

We next assessed the proportion of lysogen MAGs for bacterial families for which at least 30 near-complete MAGs (completeness >95%) were obtained. Interestingly, the proportion of lysogens in bacterial families varied from 26 to 96% of the MAGs, with families such as *Coriobacteriaceae, Eggerthellaceae, Veillonellaceae* and *Burkholderiaceae* having a low proportion of lysogens (<50% of the family’s near-complete MAGs). On the other hand, a very high prevalence of lysogen MAGs were observed for *Oscillospiraceae, Enterococcaceae,* and *Enterobacteriaceae* (>90% of the family’s near-complete MAGs) (Figure 3A). Interestingly, the observed proportion of lysogens in bacterial families did not correlate with the average relative abundance of that bacterial family in samples (Supplementary Figure 3).

The proportion of lysogen MAGs was globally consistent across bacterial families when considering the “Early,” “Late” infants and parental MAGs groups independently (Figure 3B). Notably, for several bacterial families such as *Tannerellaceae, Ruminoccoccaceae, Lachnospiraceae, Bifidobacteriaceae* and *Bacteroidaceae*, a gradual decrease in lysogen proportion was observed from “Early” infant to “Late” infant to parental MAGs. As an example, the “Early” infant MAGs for the *Bifidobacteriaceae* family had a lysogen proportion of 75% (*n* = 27), which decreased to 62% of lysogen in the “Late” infant MAGs (*n* = 31) and to 53% in the parental MAGs (*n* = 26). For the *Bacteroidaceae* family, the lysogen proportion reached 85% in the “Early” infant MAGs (*n* = 47), which decreased to 82% of lysogen in the “Late” infant MAGs (*n* = 58) and further to 65% in the parental MAGs (*n* = 50). Importantly, for the aforementioned bacterial families, the genus and species found in “Early,” “Late” infant and parental samples are distinct, reflecting the gut microbiota maturation. Focusing on species for which sufficient MAGs could be assembled from at least “Late” infant and Parental samples, we observed a decreased lysogeny proportion in parental MAGs compared to infant MAGs for most considered species, including *Phocaecola vulgatus, Bacteroides uniformis, Parabacteroides distasonis, Lachospira rogosae and Faecaibacillys intestinalis*. Other species such as *Akkermansia muniphila* and *Bifidobacterium longum* had a consistent lysogenic proportion and only *Fusicatenibacter saccharivorans* demonstrated an increased lysogeny proportion in adult MAGs compared to infant MAGs (Supplementary Figure 4).

We finally examined the potential fitness cost of lysogeny by comparing the relative abundance of lysogen and non-lysogen MAGs in their respective samples. For all investigated bacterial families, for which at least 5 lysogenic and 5 non-lysogenic MAG were obtained, the relative abundance of lysogen MAGs was not significantly different that their non-lysogenic counterpart (Supplementary Figure 5).

### 3.3. Prophages found in human intestinal gut communities are highly diverse and novel

**Figure 4:**
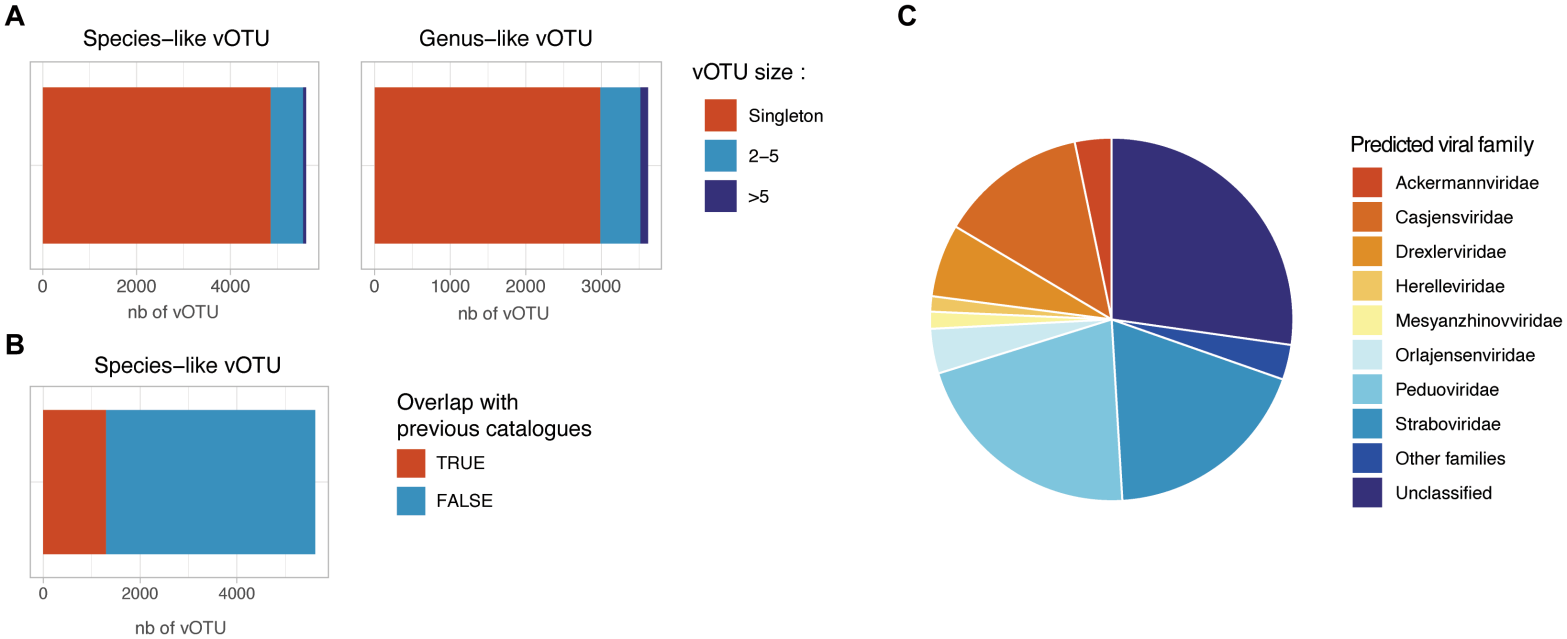
Redundancy and novelty of the identified prophage sequences. **(A)** Number of species-like and genus-like vOTUs. Species-like vOTUs were obtained by clustering sequences at 95% ANI over 75% of the shortest sequences. Species-like vOTUs were further grouped into genus-like vOTU using vContact2. **(B)** Overlap between the HELMi prophage sequences and previously published human gut phage sequences. The HELMi prophage sequences were clustered into species-like vOTU with 239,202 species-like vOTUs previously published in five bacteriophage catalogues and databases. **(C)** Predicted phage families. Representative sequences from the species-like vOTUs were classified into the new ICTV phage families using PhaGCN. Families with less than 50 sequences were grouped as “Other families.”

In order to assess the prophage diversity revealed in this study, the prophage sequences were dereplicated at 99% ANI to remove redundancy and were clustered into 5,616 species-like vOTUs using a cutoff of 95% ANI over 75% of the shortest sequence. These species-like vOTUs were clustered into 3,618 genus-like vOTUs using gene-sharing profiles based on amino-acid identity (AAI) (Bin Jang et al., 2019). Strikingly, 86.5% of the species-like and 82.5% of the genus-like vOTU generated were singletons, suggesting a low redundancy of the retrieved prophage sequences in this project (Figure 4A). Among the 846 non-singleton species-like vOTU, only 255 (30%) clustered together a prophage from infant and parental samples, suggesting a specific prophage composition in infant gut compared to adult samples. For the 759 species-like vOTU with more than one available sequence, we assessed the number of different bacterial host associated. Only 247 species-like vOTU (4.4%) were found associated with more than one bacterial species, and only 20 species-like vOTU (0.3%) were found to be associated with more than one bacterial family, suggesting a possible binning error for these vOTU.

The species-like and genus-like vOTU obtained in this project were compared to five previously published phage catalogues and reference databases derived from human stool samples from adults and children (*n* = 239,202 species-like vOTUs) (Gregory et al., 2020; Benler et al., 2021; Tisza and Buck, 2021; Unterer et al., 2021; Van Espen et al., 2021; Nayfach et al., 2021b; Nishijima et al., 2022; Shah et al., 2023; Camargo et al., 2023a). Only 1,313 of 5,616 HELMi species-like vOTUs (23%) could be clustered with a previously published species-like vOTU (Figure 4B). Surprisingly, the proportion of species-like vOTUs clustering with previously published sequences were similar when considering vOTUs retrieved found only in infant samples (22%) or parental samples (25%).

Using Genomad, 5,252 species-like vOTU (94%) were be classified as Caudoviricetes. Additionally, using PhaGCN, the species-like vOTU were classified to the ICTV viral families. This approach allowed to classify 4,093 species-like vOTUs (72.9%) into viral families (Figure 4C), with the most abundant families being Peduoviridae (21.1%) and Straboviridae (18.7%). Importantly, 1,533 (27.1%) prophage sequences could not be assigned to a known viral family by PhaGCN. The viral family proportion observed was similar when considering species-like vOTU identified in infants or in parental samples (Supplementary Figure 6).

### 3.4. Functions carried by prophages suggest a role in the modulation of bacterial host metabolism

**Figure 5:**
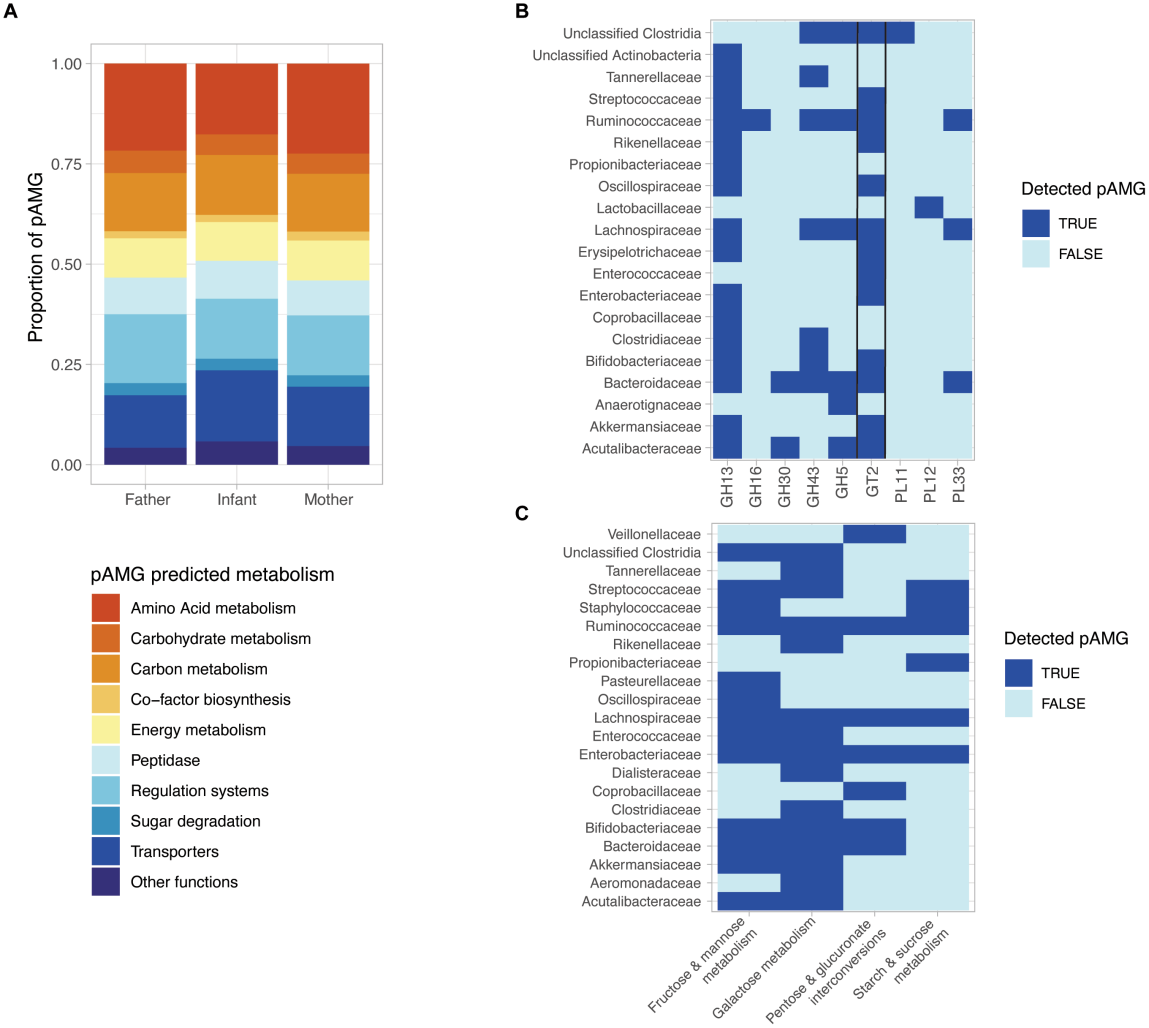
The detected prophages encode diverse putative auxiliary metabolic genes (pAMGs) involved in amino acid, nucleotide, carbohydrate, and secondary metabolite metabolism. **(A)** High-level overview of the potential metabolic role of the pAMG detected. pAMGs were predicted using DRAM-v on prophage contigs longer than 10 kb and pAMGs located between at least two phage predicted genes and at more than 5 kb from the contig ends, and without any detected transposon detected at proximity. **(B)** pAMG encoding glycosyl hydrolases (GH), glycosyl transferases (GT) and polysaccharide lyases (PLs). The pAMGs of interest were further confirmed by manually verifying their genomic context (presence of predicted phage genes upstream and downstream of the pAMG), and for concordant conserved regions and active sites in order confirm their predicted function using hhpred. **(C)** pAMG encoding enzymes for carbohydrate degradation. The pAMGs of interest were further confirmed by manually verifying their genomic context (presence of predicted phage genes upstream and downstream of the pAMG), and for concordant conserved regions and active sites in order to confirm their predicted function using hhpred.

As previously reported in a variety of ecosystems, phages can encode host genes to drive host metabolism. Putative auxiliary metabolic genes (pAMG) were predicted for the prophages sequences longer than 10 kb using DRAMv (Shaffer et al., 2020). In order to avoid potential false positives, we only considered pAMGs located between at least two phage predicted genes and at more than 5 kb from the contig ends, and without any transposon detected at proximity. These settings follow the recommendations established in Pratama et al. (2021): It is generally recommended to exclude pAMG located close to the end of a contig, to avoid a false positive detection due to an error in predicting the prophage exact boundaries. For a similar reasons, we chose to exclude putative AMGs without any upstream and downstream predicted viral gene. In total, 4,041 prophage sequences were predicted to carry at least one pAMG, accounting for 56.4% of the prophage sequences analyzed. Globally, the functions predicted for these pAMG were similar for both infant or parental derived prophage sequences (Figure 5A), with the largest proportion of the predicted metabolic function involved in amino acid metabolism (19.3%), carbon metabolism (15%) and energy metabolism (9.9%). Additionally, genes involved in transport (19.1%) or regulation systems (15.3%), were detected, but are considered as class II pAMG functions, as not involved in central metabolism (Pratama et al., 2021; Supplementary File 4).

Considering the importance of carbohydrate metabolism in the human gut microbiota, including the key infant colonizers such as *Bifidobacteria* and *Bacteroides*, we decided to further investigate the presence of pAMG predicted to encode glycosyl hydrolases (GH), glycosyl transferases (GT) and polysaccharide lyases (PLs). In order to ensure the viral origin of the investigated metabolic genes, we focused on pAMG carried by prophage sequences classified as complete or high-quality by CheckV. pAMG encoding GH, GT and PLs were manually checked for their genomic context (presence of predicted phage genes upstream and downstream of the pAMG), and for concordant conserved regions and active sites in order to ensure their predicted function. In total, 220 pAMGs could be confirmed by this method, with the most abundant pAMGs predicted to belong to the GT2 (*n* = 92), GH13 (*n* = 80), GH43 (*n* = 26), and GH5 (*n* = 11). The pAMGs were mapped to the predicted bacterial host that carried the corresponding prophage sequence. pAMGs from the GH13 and GT2 families were found in a wide range of bacterial taxa (*n* = 15 and 13 respectively), while GH5 was associated with 5 different bacterial families. MAGs from *Bacteroidaceae, Lachnospirachaceae* and *Ruminoccoccaceae* were associated to a more than 4, 5 and 6 CAZY families, respectively (Figure 5B).

We finally investigated the pAMGs predicted to encode enzymes for monosaccharide degradation, confirming the predicted hits as previously described. In total, we could confirm 129 pAMGs involved in galactose metabolism (*n* = 67), fructose and mannose metabolism (*n* = 26), starch & sucrose metabolism (*n* = 12) and pentose and glucuronate interconversions (*n* = 24). pAMG involved in galactose metabolism were carried by 15 bacterial families and pAMG involved in fructose and mannose metabolism were associated with 13 families (Figure 5C).

## 4. Discussion

Several large and curated gut virus catalogues have recently revealed the massive diversity of the human gut viral communities (Gregory et al., 2020; Camarillo-Guerrero et al., 2021; Nayfach et al., 2021b). However, most large-scale efforts focus on adult gut microbiota, and the infant gut virome remains in comparison poorly characterized. Interestingly, previous studies have shown that most of the infant virome diversity is composed of temperate phages (Liang et al., 2020; Shah et al., 2023), while adults’ gut viromes are dominated by virulent phages (Gregory et al., 2020; Brown et al., 2022). The role and importance of temperate phages have long been overlooked in virome studies as lysogenic infections leads to no apparent cellular changes in microbial communities (Howard-Varona et al., 2017) and because the identification and characterization of prophages in microbiomes remains challenging. Fortunately, the recovery of metagenome assembled genomes (MAG) from large metagenomic dataset allows the characterization of genomes from uncultured microbes (Albertsen et al., 2013). Because temperate phages can integrate in their host’s genome as a prophage, the identification of prophages in large MAG collection provides an opportunity to understand lysogeny in natural communities and better characterize host-phage interactions (Kim and Bae, 2018; Sutcliffe et al., 2023).

In this study, we explore the diversity and prevalence of integrated prophages in a 6,186 MAG catalog assembled from infant and adult fecal metagenomic samples from the broader Finnish HELMi birth cohort. Strikingly, more than 70% of the near-complete HELMi MAGs carried at least one detected prophage sequence, a prevalence significantly higher than the typical prophage prevalence reported for isolated bacterial genomes. As an example, in 2015, a total of 14,977 publicly available bacterial genomes were screened and it was estimated that 30% of the bacterial genomes contained at least one integrated prophage (Roux et al., 2015). This difference in prevalence is likely explained by the recent improvement in prophage detection tools (Schackart et al., 2023), in particular since a more recent effort observed 75% of lysogeny in the publicly available complete genomes (López-Leal et al., 2022). Importantly, a similar prevalence of prophages was previously reported in the gut microbiota of a single adult individual, where the authors reported 72% of the MAGs derived from gut bacteria were lysogen (Sutcliffe et al., 2023) Similarly, a lysogen prevalence of 70% in MAGs derived from murine gut bacteria was reported (Kim and Bae, 2018). This high prevalence of lysogeny in gut microbiota is thought to contribute to phage-mediated immunity in the gut mucosal layer (Barr et al., 2013; Silveira and Rohwer, 2016).

The Piggyback-the-Winner model suggests that lysogeny is the preferred lifestyle in dense and rapidly growing bacterial communities (Knowles et al., 2016; Brown et al., 2022). A previous study in murine gut microbiota showed a higher prevalence of lysogens in Bacillota (previously Firmicutes), and Pseudomonadota (previously Proteobacteria) compared to Bacteroidota (previously Bacteroidetes) and Actinomycetota (previously Actinobacteria), suggesting differences in growth rates among the phyla in mouse gut microbiota (Kim and Bae, 2018). Interestingly, we observed a low prevalence of lysogen for several bacterial families such as *Coriobacteriaceae, Eggerthellaceae, Veillonellaceae* and *Burkholderiaceae*, while on the other hand, MAGs obtained from *Oscillospiraceae, Enterococcaceae,* and *Enterobacteriaceae* had an extremely high prevalence of lysogen. In our dataset, the lysogeny status of the MAG was not associated to differences in terms of abundance of the taxa in the sample. However, we did not determine here if the lysogeny status was associated to difference in growth rates, as the currently available methods used for growth rate estimation have been previously shown to give spurrious results for MAGs (Long et al., 2021). Strikingly, we observed an increased lysogen prevalence in MAGs obtained from early infant samples (3 weeks and 3 months) compared to later infant samples (6 and 12 months) and adults for several bacterial families such as *Tannerellaceae, Ruminoccoccaceae, Lachnospiraceae, Bifidobacteriaceae* and *Bacteroidaceae*. This result is in line with the observation of a higher diversity and prevalence of temperate phages in infant than adult gut (Shah et al., 2023) and suggests a central role of lysogeny during the infant gut microbiota maturation.

Comparison of the HELMi prophage sequences to previously published gut phage catalogues revealed over 4,300 species-like novel vOTUs in our dataset. The observed low representation of the HELMi prophage sequences in previously identified phage sequences is consistent with the extremely large phage diversity observed in previous gut viral mining efforts (Gregory et al., 2020; Benler et al., 2021; Tisza and Buck, 2021; Unterer et al., 2021; Van Espen et al., 2021; Nayfach et al., 2021b; Nishijima et al., 2022; Shah et al., 2023; Camargo et al., 2023a). Interestingly, we also observed a high diversity of prophage sequence within the HELMi dataset and only 17.5% of the genus-like vOTUs had more than one representative sequence available. Similar to previous reports for the global (lytic and temperate phages) infant virome (Shah et al., 2023) and for prophage integrated in complete genomes available in databases (López-Leal et al., 2022), species-like and genus-like vOTUs retrieved were often specialized for a single host species, and the prophage richness largely exceeded the host richness, both at the species and genus levels.

Temperate phages have been shown to encode auxiliary metabolic genes (AMGs) that alter their bacterial host metabolism (Breitbart et al., 2007). These viral AMGs are not random but rather tuned to increase their hosts’ fitness in a specific environment (Hurwitz and U’Ren, 2016). Therefore, the characterization of viral AMGs can offer important insight into host fitness as well as the ecosystem’s nutritional constraints (Lindell et al., 2005; Hurwitz et al., 2014). In the recent years, metagenomic approaches have drastically expanded the diversity of known AMGs, including genes involved in carbon metabolism, sugar metabolism, lipid–fatty acid metabolism, signaling and stress responses, energy and nitrogen metabolism (Breitbart et al., 2018; Kieft et al., 2020; Shaffer et al., 2020; Fremin et al., 2022). In adult gut, a previous study suggested the presence of potential AMG (pAMG) encoding a large number of functions such as amino acid and carbohydrate transport and metabolism (Monaghan et al., 2019; Shaffer et al., 2020). In this study we observed a high prevalence of pAMGs encoding for amino acid metabolism as well as carbon and energy metabolisms, for both infant and adult pAMGs.

We further explored the pAMGs associated with carbohydrate metabolisms and reported pAMG encoding 5 different glycoside hydrolase families involved in the degradation of plant-based dietary polysaccharides (GH13, GH16, GH30, GH42, GH5). Importantly, pAMGs encoding glycosides hydrolases were previously reported in adult gut viromes (Shaffer et al., 2020) and in soil, where the functionality of a GH5 pAMG was experimentally verified (Trubl et al., 2018). Importantly, the functional role of these glycoside hydrolases has been suggested to include also adhesion of the virion to bacterial cells or host-associated mucosal glycans (Benler et al., 2021; Rothschild-Rodriguez et al., 2023). We also detected the presence of phage encoded glycosyltransferases from the GT2 family that has been shown to be able to confer protection to phage infection by modifying the bacterial capsule polysaccharide structure (Porter et al., 2020). This result suggests a potential defense system encoded by integrated phages, in which the integrated prophage could benefit their host by limiting phage co-infection. While the true role and nature of these pAMG and their potential impact on their host fitness, microbial ecology and microbiota-host interaction remains to be determined, these phage genes are of particular interest, as these genes may expand the metabolic repertoire of the bacterial host.

## 5. Limitations and future directions

MAGs provide a new opportunity to investigate the diversity and prevalence of prophages directly in complex ecosystems. However, one of the main limitations of this approach is that high-quality MAG reconstruction is only possible for the most abundant genomes in each sample, therefore limiting the type of phage-bacteria interactions that can be investigated. Moreover, this approach could lead to some false positive prophage association to bacterial host due to errors in the binning process. We tried to limit these false associations by restricting our study to prophage sequences that were assembled with sequences from the bacterial host genome, but only complete sequencing from isolated bacteria would allow to ensure the definitive presence of an integrated prophage in these genomes. It is important to note that by using this conservative approach, we certainly underestimated the true proportion of lysogens. Furthermore, while phage detection tools have recently greatly improved, it cannot be excluded that a proportion of integrated prophages in our dataset were missed in our analysis. Altogether, it is highly probable that our analysis is an under-representation of the true proportion of lysogens found in human guts.

Additionally, prophage decay, during which prophages lose genes, including those necessary for virion production (Bobay et al., 2014), was not assessed in this study. This means that we cannot exclude that a proportion of the prophages reported in this study are not inducible.

AMG identification has first been done using manual inspection of the phage genomes, but recently new automated tools allow for high throughput annotation of candidate AMGs from large phage datasets. DRAM-v leverages expert-curated AMG databases for functional annotation and provides the user with a scoring system to assess the likelihood of the AMG prediction (Shaffer et al., 2020). In this study, we used a strict quality threshold to identify high-confidence virus sequences, and only took into account sequences longer than 10 kb to predict pAMGs. Additional verifications of the pAMGs genomic context and for concordant conserved regions and active sites was done as recommended in Pratama et al. (2021). However, it is important to note that the role of these pAMGs is largely undetermined, and we cannot therefore exclude that the observed pAMGs are not legitimate AMGs. Further experimental studies will be required to assess the true role and function of the pAMGs reported in this study.

## Data availability statement

The sequence data that support the findings of this study are available in European Nucleotide Archive under the Project ID: PRJEB52774, and in Zenodo under the archive ID: 8063476 and in the Supplementary material.

## Ethics statement

The studies involving humans were approved by Ethical committee of The Hospital District of Helsinki and Uusimaa. The studies were conducted in accordance with the local legislation and institutional requirements. Written informed consent for participation in this study was provided by the participants’ legal guardians/next of kin.

## Author contributions

ED: data curation, formal analysis, investigation, Writing – original draft, writing – review and editing. DM: data curation, formal analysis, Investigation, writing – original draft, writing – review and editing. EL: data curation, investigation, writing – review and editing, formal analysis, methodology. K-LK: resources, writing – review and editing. WD: funding acquisition, resources, writing – review and editing. AS: funding acquisition, resources, supervision, writing – review and editing. AP: conceptualization, data curation, formal analysis, funding acquisition, investigation, methodology, supervision, writing – original draft, writing – review and editing.

## Funding

This study was supported by grants from Business Finland grant 329/31/2015 to WD and AS, Academy of Finland (339172 to AP and 1325103 to AS), European Union’s Horizon 2020 Research and Innovation Program H2020 MSCA (Sweet Crosstalk) project under grant agreement no. 814102 to AS. DM acknowledges the funding for Ph.D. received through European Union’s H2020-MSCA-ITN-2018 Sweet Crosstalk project under grant agreement no. 814102.

## Conflict of interest

The authors declare that the research was conducted in the absence of any commercial or financial relationships that could be construed as a potential conflict of interest.

## Publisher’s note

All claims expressed in this article are solely those of the authors and do not necessarily represent those of their affiliated organizations, or those of the publisher, the editors and the reviewers. Any product that may be evaluated in this article, or claim that may be made by its manufacturer, is not guaranteed or endorsed by the publisher.

## Supplementary material

The Supplementary material for this article can be found online at: https://www.frontiersin.org/articles/10.3389/fmicb.2023.1254535/full#supplementary-material

## Footnotes

^1^available at https://www.ncbi.nlm.nih.gov/assembly/GCF_000001405.26/

^2^Available at https://github.com/KennthShang/PhaGCN_newICTV
