# Supplementary material for: An extended catalog of integrated prophages in the infant and adult fecal microbiome shows high prevalence of lysogeny
Source: Front Microbiol. 2023 Sep 5;14:1254535. doi: 10.3389/fmicb.2023.1254535 (PMC10508911; doi:10.3389/fmicb.2023.1254535)
Supplement: Supplementary file 1 [file Data_Sheet_1.zip › Supplementary Tables and Figures.pdf]

Supplementary Material

Supplemental Table 1

A. Infant characteristics

Sex at birth:	**Male**	**Female**
n	44	44
Vaginal delivery, n (%)	32 (72%)	32 (72%)
Received Intrapartum antibiotics, n (%)	27 (61%)	20 (45%)
Intensive care at birth, n (%)	6 (14%)	4 (1%)
Weight at birth in g, median (IQR)	3542 (613)	3551.5 (501.5)
Height at birth in cm, median (IQR)	50.5 (2.25)	50 (2)

B. Parental characteristics

Individual:	**Pregnant Mother**	**Father**
n	68	70
Age, median (IQR)	32 (4)	34 (6)
University education level, n (%)	44 (65%)	37 (53%)
Distance in days from sample collection to delivery, median (IQR)	-8 (8)	-5 (7.75)
Pregnancy duration in days, median (IQR)	283.5 (10.25)	
Diagnosis of gestational diabetes, n (%)	11 (16%)	
Parity, n (%)	23 (34%)	

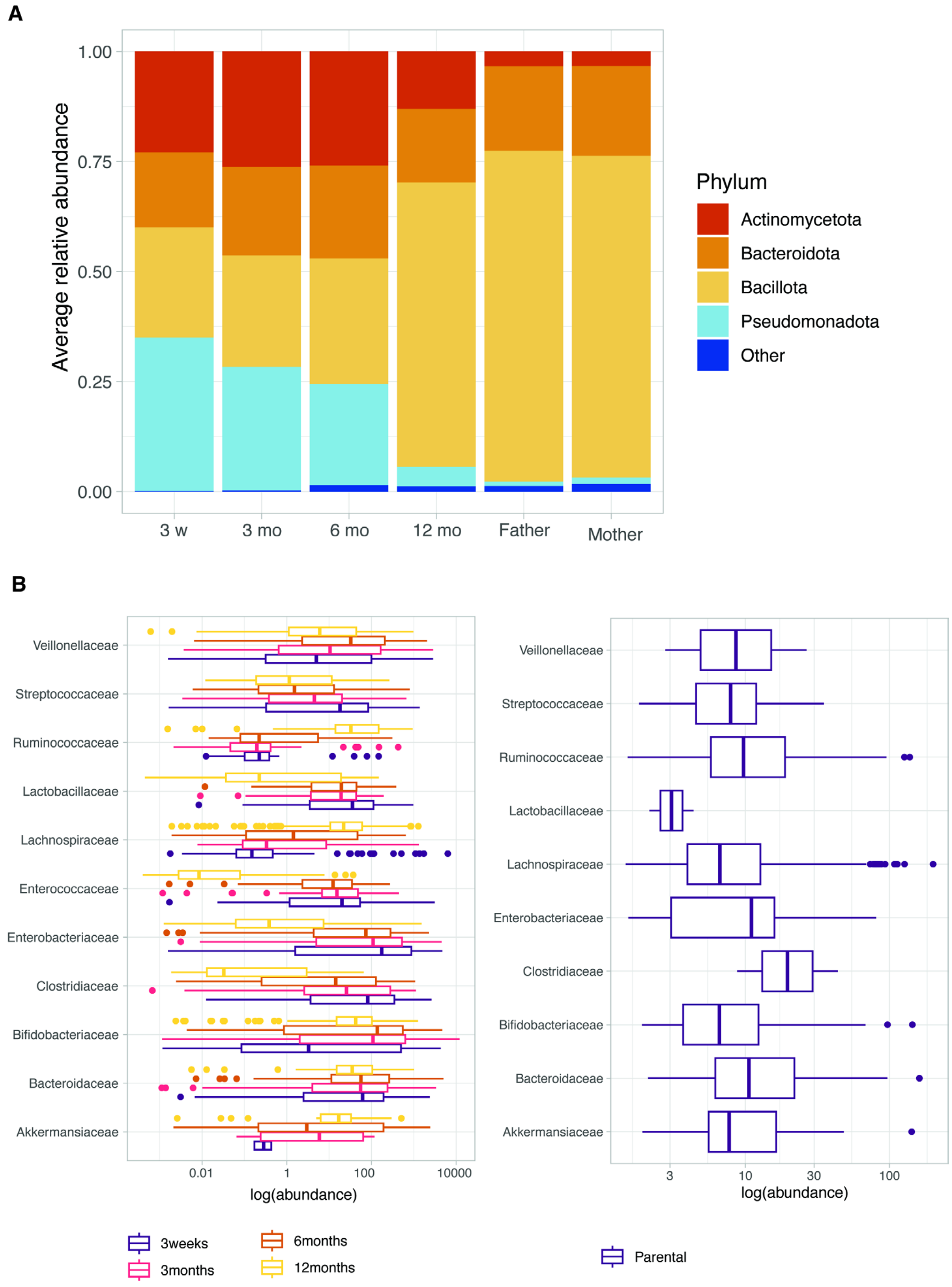

Supplemental Figure 1: Read-based average composition and MAG abundance in samples. (A) Average relative abundance of bacterial phylum for infants and parental samples grouped per sampling age. Quality controlled reads were taxonomically annotated using Kraken2 and Bracken against the HumGut database and counts were aggregated at the phylum level.

(B) Abundance of MAGs in their respective samples, grouped per bacterial families and sample age. The abundance of each MAG was calculated as genome copies per million reads.

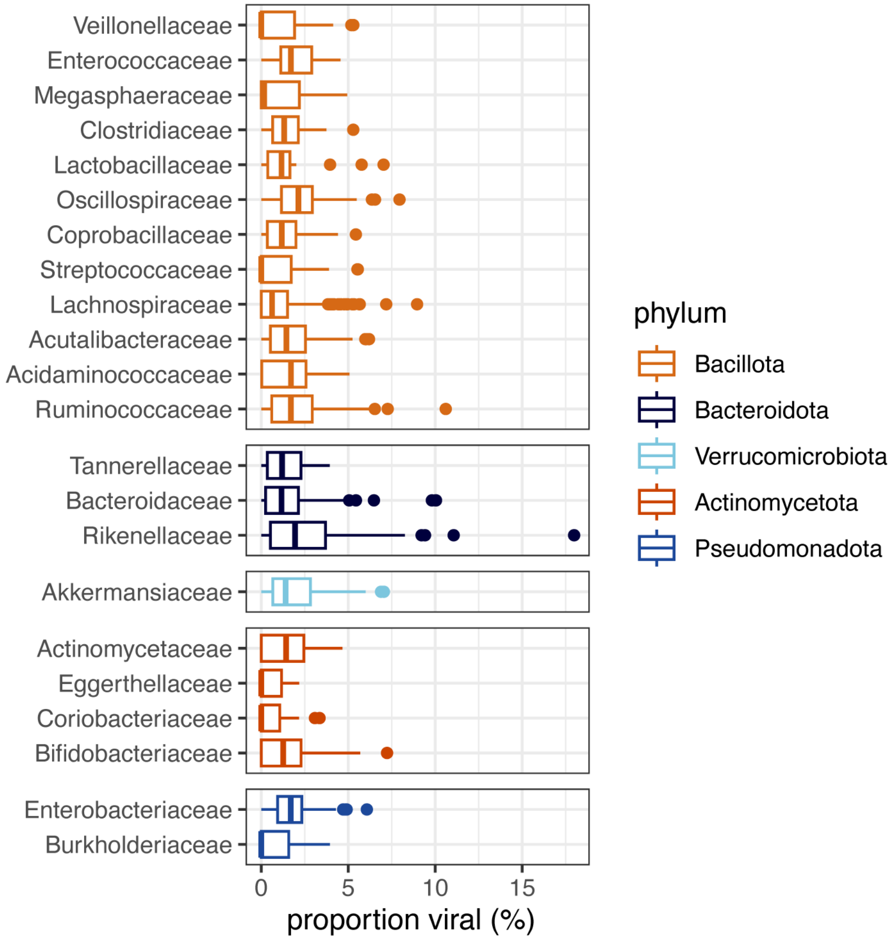

Supplemental Figure 2: Ratio between MAG length and prophage sequence length by bacterial families. MAGs were sub-setted to include only high-quality MAGs (above 95% completeness), and only families for which more than 20 MAGs were retrieved were included.

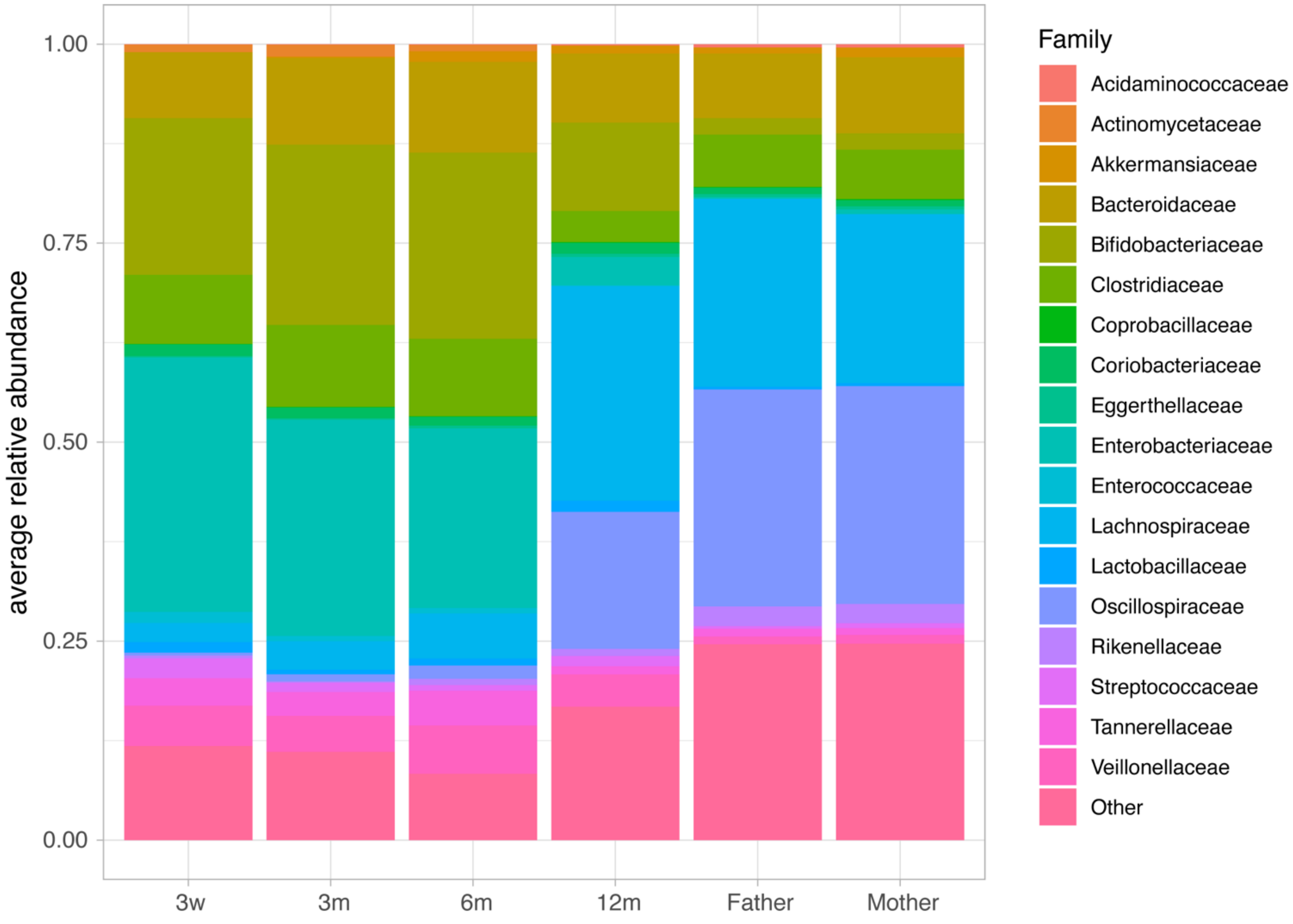

Supplemental Figure 3: Read-based average composition at the family level Average relative abundance of bacterial families for infants and parental samples grouped per sampling age. Quality controlled reads were taxonomically annotated using Kraken2 and Bracken against the HumGut database and counts were aggregated at the family level.

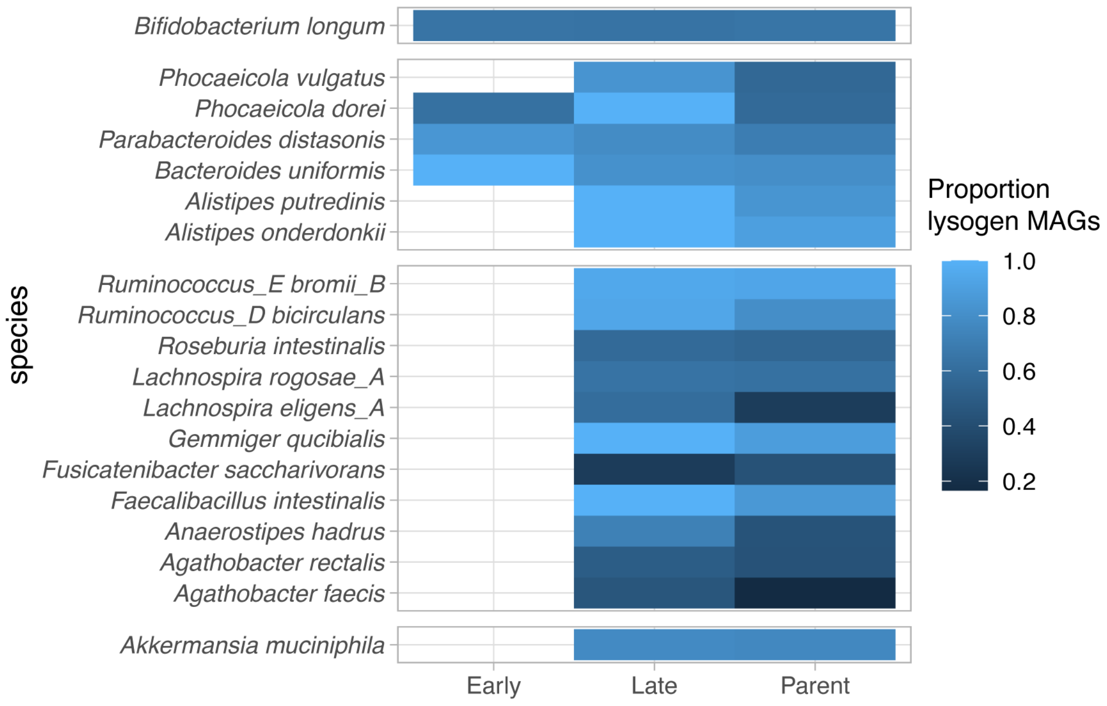

Supplemental Figure 4: Lysogeny prevalence in infant and adult species MAGs.
Proportion of lysogen MAGs per bacterial species and MAG groups. The MAGs assembled from infant samples were categorized into "Early" and "Late" categories according to their relative abundances at early (3 weeks and 3 months) and late (6 and 12 months) sampling time points. We selected bacterial species for which more than 5 near-complete MAGs (>95% completeness) were available in parental and one infant MAG group. The proportion of lysogen MAGs (MAG with at least one detectable prophage sequence) was calculated independently per bacterial. Bacterial families are grouped by phylum.

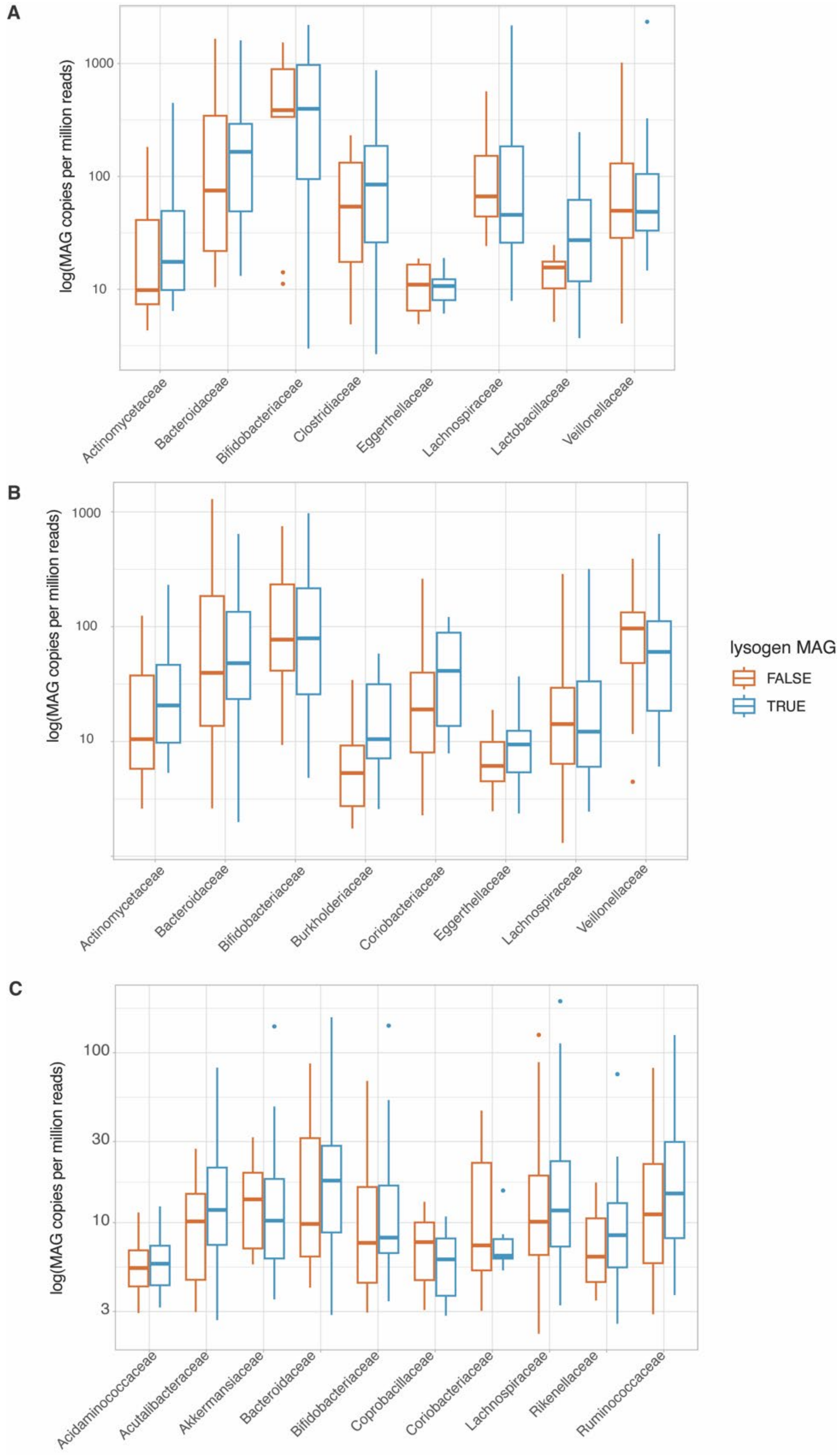

Supplemental Figure 5: Relative abundance of lysogenic and non-lysogenic MAGs in samples

Abundance of MAGs in their respective samples, grouped per bacterial families in (A) “Early” infant samples (3weeks and 3months), (B) “Late” infant samples (6 and 12 months) and (C) in adult samples. The abundance of each MAG was calculated as genome copies per million reads.

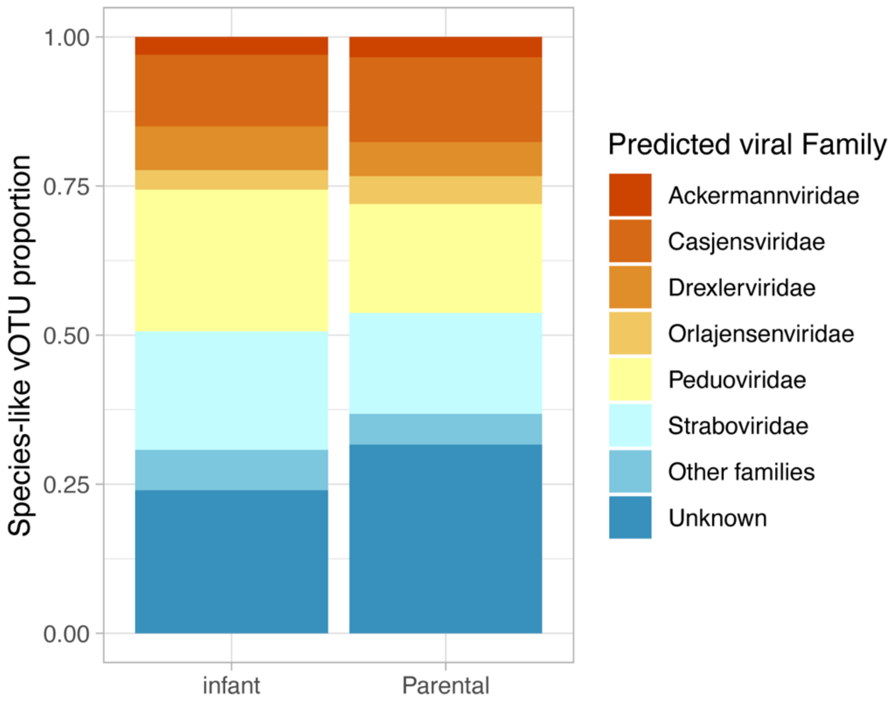

Supplemental Figure 6: Predicted phage families in infant and parental samples.

Representative sequences from the species-like vOTUs excluding vOTUs with representative sequences isolated from both parental and infant MAGs, were classified into the new ICTV phage families using PhaGCN. Families with less than 50 sequences were grouped as ”Other families”.
